# Novel Porcine Circoviruses in View of Lessons Learned from Porcine Circovirus Type 2-Epidemiology and Threat to Pigs and Other Species

**DOI:** 10.3390/v14020261

**Published:** 2022-01-27

**Authors:** Hanna Turlewicz-Podbielska, Agata Augustyniak, Małgorzata Pomorska-Mól

**Affiliations:** Department of Preclinical Sciences and Infectious Diseases, Poznan University of Life Sciences, Wolynska 35, 60-637 Poznan, Poland; hanna.turlewicz@up.poznan.pl (H.T.-P.); agata.augustyniak@up.poznan.pl (A.A.)

**Keywords:** porcine circovirus type 3, porcine circovirus type 2, pathogenicity, cross-species transmission

## Abstract

Porcine circovirus type 2 (PCV2) plays a key role in PCV2-associated disease (PCVAD) etiology and has yielded significant losses in the pig husbandry in the last 20 years. However, the impact of two recently described species of porcine circoviruses, PCV3 and PCV4, on the pork industry remains unknown. The presence of PCV3 has been associated with several clinical presentations in pigs. Reproductive failure and multisystemic inflammation have been reported most consistently. The clinical symptoms, anatomopathological changes and interaction with other pathogens during PCV3 infection in pigs indicate that PCV3 might be pathogenic for these animals and can cause economic losses in the swine industry similar to PCV2, which makes PCV3 worth including in the differential list as a cause of clinical disorders in reproductive swine herds. Moreover, subsequent studies indicate interspecies transmission and worldwide spreading of PCV3. To date, research related to PCV3 and PCV4 vaccine design is at early stage, and numerous aspects regarding immune response and virus characteristics remain unknown.

## 1. Introduction

Circoviruses belong to the *Circoviridae* family, genus *Circovirus* [1]. They are the smallest autonomously replicating, non-enveloped, single-stranded DNA viruses with circular symmetry [2]. Circoviruses were originally described as avian species, but some of them have been identified in fish, insects and mammals [3,4]. Until now, four species of circovirus are known to infect pigs, including porcine circovirus (PCV) 1, PCV2 and the novel PCV3 and PCV4 [5,6,7,8,9].

The genome size of porcine circoviruses varies depending on the species of circovirus, ranging from 1758nt to 2001nt [10]. The genome contains two major ORFs, located on opposite strands, ORF1 and ORF2, which encode replication-related proteins—Rep and Rep’—and the capsid protein (Cap), respectively [11]. PCV1 was first described in 1974 and proved to be non-pathogenic [5,12]. PCV2 was identified in 1998 as the causative agent of post-weaning, multisystemic wasting syndrome (PMWS) in western Canada [6]. The last two representatives were discovered more recently. PCV3 was identified in 2016 in pigs with symptoms similar to porcine dermatitis and nephropathy syndrome (PDNS), together with reproductive disorders [7], and in animals with symptoms of multisystemic disease and myocarditis [8]. PCV4 was reported in 2019 in the Hunan province of China in pigs with PDNS-like symptoms and severe symptoms in respiratory and digestive tracts [9]. 

Since ~2000, there has been a significant intensification of swine husbandry, contributing to an increased number of viral infections in swine and the emergence of new pathogens. PCV2 plays a significant role in the economics of pig husbandry, leading to great losses in the pig industry in the last 20 years and a key role for PCV2-associated disease (PCVAD) etiology. The increased mortalities and the decrease in daily gains generate a large amount of additional costs, which include processing the carcasses, additional feed, veterinary care, drugs, greater consumption of electricity, water and straw [13].

On the other hand, newly emerged PCVs, like PCV3, may potentially contribute to greater economic losses than PCV2 due to easy cross-species transmission and spreading as well as a potential high evolutionary rate [14]. Controlling novel PCVs is critical to diminishing the possibility of the spread of various genotypes in the future. Due to the PCV3 isolation from pigs with reproductive problems, a link between PCV3 and reproductive failure is likely. Retrospective studies of PCV2 showed that PCV2 was present in the pig population before serious clinical problems appeared [15]. As with PCV2, the susceptibility of pigs to PCV3, the pathomechanism of PCV3 infection and other factors can change and make PCV2 infection more severe. Active surveillance, further molecular and epidemiological studies as well as initial vaccine research are advisable in the view of numerous knowledge gaps related to PCV3 and PCV4 structure and pathogenesis. In the case of PCV4, it is too early to know if it is clinically significant in swine husbandry.

## 2. Occurrence and Spread of New Porcine Circoviruses

PCV3 was initially detected in 2016 in pigs in North America and Asia and then confirmed in European countries; over time it spread to South America and Africa [7,16,17,18,19]. The virus was isolated from porcine serum, tissues (lymph nodes, lungs), oral fluid, and nasal swabs [7,20,21]. In China, the occurrence of genetic material of PCV3 was confirmed in aborted fetuses, semen and serum from pigs with symptoms of reproductive disorders, including massive abortion [16].

Recent results indicate that PCV3 is widespread in Asia [16,20,22,23,24]. It was confirmed in 77 (34.7%) of 222 samples and in 24 (68.6%) of 35 farms in China in 2017 [16] and in 72 (36.36%) of 198 samples collected in 2021 [22]. In 2017, DNA of PCV3 was identified in 159 (44.2%) of 360 swine oral fluid samples from 73 farms in South Korea [20]. In Japan, 6 (22.2%) of the 27 farms tested and 7 (9.6%) of the 73 samples were positive for PCV3 [23]. In Thailand, PCV3 was confirmed in 5 (62.5%) out of 8 lung and lymph node samples collected from pigs [24]. The virus has been confirmed also in Russia at two farms at significantly distant locations (Smolensk, in the western part of Russia, and Tyumen, in the eastern part) [25]. The virus was also confirmed in India and detected in tissue samples collected from aborted fetuses as well as serum and nasal swabs from sows [21]. The latest reports on PCV3 detection concern Taiwan, where a retrospective analysis of samples collected from clinically symptomatic pigs between 2016 and 2019 revealed PCV3 DNA in 7 (10.6%) out of 66 samples in 2016, 22 (12.64%) out of 174 samples in 2017, 36 (33.33%) out of 108 samples in 2018 and 40 (34.78%) out of 115 samples in 2019 [26], and Malaysia, where 24 (17.02%) of 141 tested pigs were PCV3 positive [27].

In the USA, PCV3 was determined for the first time in 2016 in samples from pigs with PDNS-like symptoms together with reproductive disorders [7] and in animals with symptoms of multisystemic disease and myocarditis [8]. In 2017, PCV3 was detected in Brazil in serum samples from sows that had aborted [18]. PCV3 was recently confirmed in Colombia [28] and Argentina [29].

**Figure 1:**
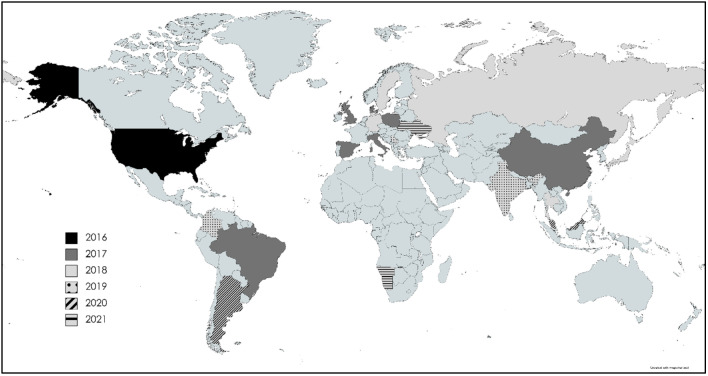
The worldwide occurrence of PCV3.

PCV3 has also been identified in Europe. The presence of PCV3 was confirmed in pigs in Poland. The samples collected from pigs with varying health status reared in 14 farms located in different regions of the country were included in the analyses; 85.7% of the farms were positive [17]. Soon after, it was detected in the tissues of aborted fetuses in Italy [30] and tissue and serum samples from pigs in Denmark and Spain [31], as well as in feces and tissues collected from pigs from the UK [32]. In Sweden, PCV3 was identified in 10 (20.41%) of 49 lymph nodes collected from pigs between 1993 and 2003 [33]. It is worth mentioning that one of the positive samples was collected in 1993, showing that PCV3 has been circulating in Sweden for a long time [33]. In Germany, the virus was confirmed in 40 (75%) out of 53 tested farms [34]. PCV3 in pigs was also confirmed in Hungary [35]. The genetic material of PCV3 was detected in 5 (15.6%) out of 32 serum pools and in 28 (84.4%) out of 33 tissue pools collected in Serbia [36]. In Ukraine, PCV3 was confirmed in clinically healthy pigs originating from two farms in the Kyiv and Kharkiv regions [37]. In 2021, PCV3 was confirmed in Africa (Namibia) for the first time [19]. Subsequent studies indicate that PCV3 is spreading worldwide (Figure 1).

Despite PCV3 having been discovered relatively recently, retrospective studies indicate that it has been circulating in the swine population for many years. The oldest sample in which PCV3 was identified is from 1967 from Brazil [38]. Slightly younger ones come from Sweden in 1993 [33] and from China and Spain in 1996 [39,40].

**Figure 2:**
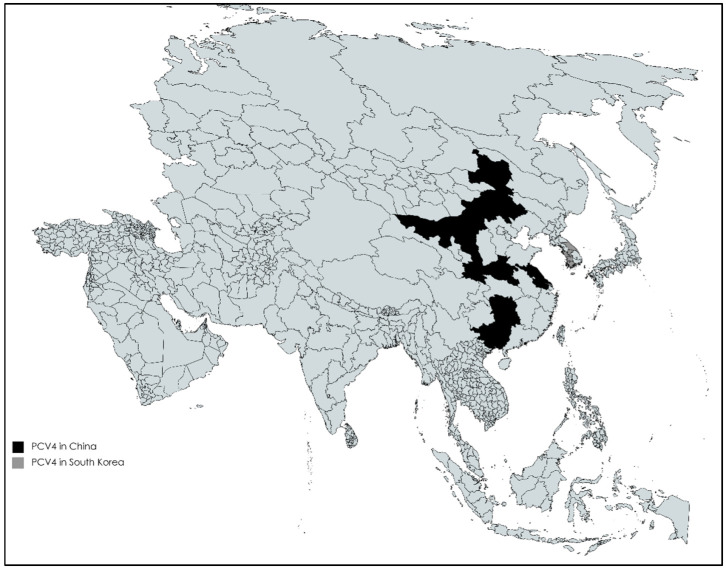
The occurrence of PCV4 in Asia.

PCV4 has been discovered recently in samples (serum, lungs, kidneys, spleen) obtained from pigs with clinical symptoms, including respiratory symptoms, diarrhea, skin lesions and systematic disease, from two farms located in Hunan province, China. A total of 187 clinical samples from pigs were investigated, and the overall PCV4 prevalence was 12.8%, with the highest positive rates in nasal swabs; 6 (28.5%) out of 21 samples were PCV4 positive [9]. However, research conducted in 2021 indicates that PCV4 has been circulating in this country for up to ten years [41]. Retrospective analysis of tissue and serum samples obtained from 2011 to 2021 from 49 different farms in 15 cities in the Henan province revealed that PCV4 has been circulating in this area since at least 2012 [41]. Since then, several new reports on the detection of this virus in other provinces of China appeared: Guangxi, where 13 (5.1%) of the 257 tested samples were positive [42], Henan and Shanxi, in which 16 out of 63 samples were positive (25.4%) [43], Inner Mongolia, where the percentage of positive samples was 1.6% (27/1683) [44] and Jiangsu, where 4 (3.33%) of the 120 tested samples were PCV4 positive [45]. The only country outside of China where PCV4 has been reported is South Korea (Figure 2). It was determined in 6 out of 9 regions, and the mean prevalence was 3.28% (11/335) [46]. A total of 108 serum and tissue samples from Spain and 163 serum and tissue samples from Italy from both diseased and clinically healthy animals were tested, although PCV4 DNA was not detected [47].

## 3. Diversity and Evolutionary Rate of Porcine Circoviruses 

To date, eight PCV2 genotypes (a to h) have been described; however, the evolution is still ongoing, and new genotypes are evolving. The first two genotypes (a, b) were classified in 2007 based on differences in the ORF2 nucleotide [48]. In 2009, during research on isolates of this virus, three more genotypes, named as PCV2c, PCV2d and PCV2e, were described [49]. PCV2d was also identified a year later by sequence analysis of additional amino acids from its capsid protein gene, resulting from a mutation in the stop codon of ORF2 [50]. The current classification of PCV2 genotypes is based on the following criteria: maximum intra-genotype p-distance of 13% (calculated on the ORF2 gene), bootstrap support at the corresponding internal node higher than 70% and at least 15 available sequences [51]. In 2018, researchers from the US suggested distinguishing the PCV2i genotype [52]. Currently, three genotypes dominate in the world: a, b and d [51]. The other genotypes are documented rather sporadically.

Since the identification of PCV2, there have been changes in genotype prevalence, known as “genotype shifts”. There is a clear genotype shift towards genotypes b and d. In the mid-2000s, the initially predominant genotype of PCV2a was replaced by PCV2b. In recent years, the incidence of the PCV2d genotype has increased in many regions around the world [53,54]. At present, cross-reaction between different PCV2 genotypes is sufficient, and there is no need to update the vaccines, although it should be kept in mind that the status quo may change.

Mutations in the sequences encoding the immunogenic capsid protein may explain the direction of PCV2 genotypic evolution. Wei et al. (2019) performed phylogenetic analysis on 43 PCV2-positive samples collected in Belgium from 2009 to 2018 and demonstrated PCV2 evolution toward a better receptor binding capacity. In this study, mapping of the PCV2 Cap-specific amino acids revealed that most of the mutations were located outside of Cap. Mutations on the upper and tail parts of the Cap evolved towards efficient and stronger binding to glycosaminoglycan receptors, probably to escape from the host immunity [55]. Porcine circoviruses show considerable variability and evolve rapidly, which is not typical for DNA viruses. A high substitution rate in single stranded DNA in PCV2 allows evolutionary dynamics similar to single stranded RNA viruses [56,57] and represents a substrate for selective pressure occurrence. Selective, immune-originating pressure deriving from natural infection and vaccine-induced responses influences the variability through the capsid structure and plays a key role in progressive evolution, affecting PCV2 strains, genotypes and spreading of the virus [57]. PCV2 vaccines are based on PCV2a and limit the prevalence of PCV2a, albeit they yield increased diversity among the PCV2a genotype group, mainly focused at PCV2a epitopes shared by vaccine and field strains [53]. However, the first genotype shift from PCV2a to PCV2b was noted during the period of 2003–2004, before implementation and registration of the PCV2a-type commercial vaccine in 2007. This genotype evolution was probably related to the infection immunity, common in all farms [55]. As mentioned earlier, the high frequency of natural infections may also contribute to the genetic evolution. PCVs can spread easily within the population through horizontal transmission by secretions and excretions of infected pigs, as well as vertical transmission [58].

Among new PCVs, three major genotypes of PCV3 are identified with the use of phylogenetic methods: PCV3a, PCV3b and PCV3c [59]. To date, the prevalence, geographic distribution and potential differences in the virulence of these genotypes are unknown. 

Li et al. (2018) suggest the substitution rate of PCV3 is higher than in PCV2, making the novel circovirus privileged to adaptation to various biological conditions [60]. When PCV3 strains establish in a specific area, they can likely adapt to the local host [60]. Amino acid site 24 of the ORF2 protein (codon of 320 in the complete coding region) is believed to be a potential epitope, which is under positive selection. It may be a pivotal trigger for host immune system evasion, providing prolonged circulation and the divergence of the PCV3 genotypes [60].

On the other hand, researchers point out that PCV3 strains isolated over the years show relatively high genetic stability, which confirms their high similarity [61,62]. Despite the fact that they belong to different clusters, the nucleotide identity among these sequences is genuinely high (>97%). This shows that PCV3 has remained rather stable over the years, and independent molecular evolution in specific areas of the world did not occur [61]. Based on high-quality and updated sequence data, including sampling times greater than 20 years (1996–2018), Franzo et al. (2019) showed a low PCV3 mutation rate (i.e., ≈10−5 substitutions/site/year). The authors even suggest that the origin of PCV-3 could have been centuries ago [63]. These results are consistent with the assumption of limited genetic variation and high similarity between the last sequences and those obtained in the mid-1990s [61]. A potential underestimation of the most recent common ancestor of PCV-3 cannot be ruled out on the basis of PCV3’s great diversity compared to other known circoviruses. Nevertheless, the issues related to the mutation frequency of PCV3 remain controversial, and more studies on the evolution of the virus are required. Further improvements in mathematical modeling capabilities may contribute to address this controversy. 

## 4. Porcine Circoviruses as a Potential Threat to Other Species

PCVs cross-species transmission is likely to be a serious threat to the global pig industry and other animal industries. The contamination of feed with PCV-infected chicken and swine products is a potential source of infection to farm animals [64]. PCV2 was detected in bone marrow, blood and brain samples of humans, mice, cows and calves [65,66,67,68]. This virus was also determined in *Musca domestica* flies and culex mosquitoes in pig farms [69]. Recently, the first emergence of PCV2 in raccoon dogs and foxes was documented [70,71]. In racoon dogs, the PCV2-positive rates were 89.3% (*n* = 150) in animals with symptoms of abortion or sterility and 37.3% (*n* = 150) in healthy animals [70]. The PCV2-positive rate was also higher in aborted or sterile animals compared to healthy foxes. The PCV2-positive rates were 94.8% (*n* = 135) in diseased foxes and 9.3% (*n* = 150) in foxes with no symptoms [71]. The results of these studies indicate that PCV2 was probably associated with reproductive failure in raccoon dogs and foxes. The similarity of six raccoon dog–origin PCV2 strains among 40 PCV2 representative strains varied from 92.1 to 99.8% [70]. Three PCV2 strains detected in foxes shared a similarity of 91.9 to 99.7% with nucleotide sequences of 41 representative PCV2 strains published in GenBank. Strains isolated from racoon dogs and foxes belonged to Chinese epidemic genotypes PCV2b and PCV2d and could be transmitted from pigs to raccoon dogs and foxes. PCV2 was also isolated from tissues of minks that died from diarrhea [64]. The genome of PCV isolated from minks had 84.25–99.77% similarity with PCV2 and was distantly related to mink circoviral species, which indicates the cross-species transmission [64].

The emergence of PCV3 is believed to be the result of intensive farming development [72]. As expected, this virus circulates with high prevalence among wild boars [72,73,74,75,76,77,78]. In Klaumann (2019), serum samples from wild boars from the years 2004 to 2018 were investigated. The results revealed a high frequency of PCV3 detection (42.66%; *n* = 518) and demonstrated circulation since at least since 2004 [73]. High prevalence (61.54%; *n* = 39) of PCV3 was also noted in blood and tissue samples of Sardinian wild boars [74]. Wild Suidae can act as an important reservoir of PCV3. This species was confirmed in wild boars from Brazil, Germany, Spain, Italy and Korea [40,73,74,75,76,77,78]. Czyżewska-Dors et al. (2021) detected PCV-3 DNA in sera of wild ruminants in Spain: red deer (*Cervus elaphus*) (0.9%; *n* = 108), mouflon (*Ovis aries*) (1.1%; *n* = 91), and fallow deer (*Dama dama*) (0.96%; *n* = 104). Epidemiological cycles involved in transmission and maintenance of this virus in wildlife are unknown and require further investigation [79]. A recent study from Italy reports the tick species *Ixodes ricinus,* collected from several species of wild ungulates from different mountain areas of Friuli Venezia Giulia, Italy, (*Rupicapra rupicapra, Cervus elaphus, Ovis musimon, Capreolus capreolus, Sus scrofa*), was positive for PCV3 in real-time PCR [72]. Importantly, viral genome detection in ticks does not prove that ticks may act as a PCV3 vector for wildlife animals; however, this possibility should not be neglected. PCV3a and PCV3b DNA was also determined in 74 (34.74%; *n* = 213) serum samples collected from cattle without clinical symptoms in China [80]. Lately, Wang et al. (2021) reported PCV3 DNA in blood samples of female donkeys presenting reproductive disorders. Positive rates of PCV3 reached 21% all 300 samples from female donkeys with reproductive failure [81]. However, the authors pointed out that the association of PCV3 infection and reproductive failure in donkeys is not completely understood. Zhang et al. (2018) detected PCV3 DNA in the serum of four (9.09%; *n* = 44) samples from dogs. All animals in this study were negative for PCV2 and canine circovirus. The investigated dogs presented various clinical signs (respiratory, enteric, etc.) and were sampled during their medical checkups by veterinarians from veterinary hospitals in Hunan province, southern China [82]. According to Palinski et al. (2017), PCV3 has a close evolutionary relationship with canine circovirus [7]. Moreover, dogs infected with canine circovirus had hepatic pathology [83] similar to sows infected with PCV3 (necrotizing vasculitis and granulomatous lymphadenitis) [84]. Zhang et al. (2018) suggest that PCV3 may be propagated in hosts other than pigs, although the pathogenicity of this virus in dogs has not been determined [82]. PCV3 was also detected in laboratory mice in China. All 20 samples from mice were positive for PCV3 DNA, indicating the potential usefulness of this species for studies of the pathomechanism and spreading of PCV3 infection through other animal populations [85].

Franzo et al. (2018) hypothesized that PCV3 might be a product of the recombination of a mammalian-virus (likely a bat-circovirus) Rep gene with an avian circovirus-like Cap gene and is related to a host jump. The presence of quite distinctive patterns in genomic composition, dinucleotide frequency and codon bias between circoviruses infecting mammalian and avian species was observed during analysis of 2555 Rep and 4424 Cap sequences obtained through the NCBI Taxonomy browser (accessed on 15 October 2017). Unfortunately, the number of strains collected from host classes other than Aves and Mammalia was scarce in this study, and the analyses were focused on the circoviruses infecting mammals and birds [86]. Li et al. (2018) confirmed clade 1 bat CVsm, isolated in China from 2011 to 2013, shared the most recent common ancestor with PCV3. The same research team points out PCV1 and PCV2 are closely related to the clade 2 Bat CVs [60].

Studies on PCV4 suggest that the PCV4 Cap has high identity (70%) with the Cap of mink circovirus [9], although infections of species other than the pig are unknown to date.

## 5. Zoonotic Potential of Porcine Circoviruses

**Figure 3:**
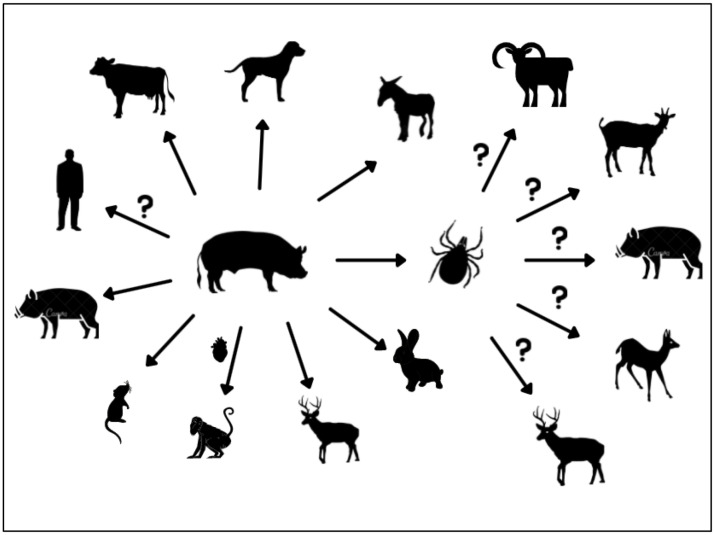
Possible cross-species transmission of PCV3. Arrows represent proven transmission of PCV3. Question marks represent possible but unconfirmed PCV3 transmission.

Circovirus host jumps may be a potential threat to public health. It is not clear if PCVs, including PCV3 and/or PCV4, have zoonotic potential at this moment. Both PCV1 and PCV2 can infect human cells; nevertheless, the infection efficiency of PCV2 is lower in human cells than in PK-15 cells, indicating PCV2 infection is limited in human cells [87]. In addition, vaccines against a rotavirus contaminated with PCV1 and PCV2 did not induce an infection in the mostly juvenile vaccinated human subjects [88]. Interestingly, Krüger et al. (2019) showed that PCV3 is able to infect non-human primates. They transplanted hearts from four of the PCV3-positive pigs into baboons. Analysis of the samples collected from baboons revealed PCV3 in all organs. Despite the fact that the attempts to infect the human kidney 293 cell line failed [89], the authors suggest that the lack of infection in this study does not exclude other human cells from being infected with PCV3. Antibodies against PCV1 or PCV2 DNA were detected in human sera, digestive tract samples and respiratory tract samples in several studies [4,90,91,92,93]. The possible cross-species transmission of PCV3, including zoonotic transmission, is illustrated in Figure 3.

## 6. Pathogenicity of New Porcine Circoviruses for Pigs

The pathogenesis of PCV3 infection is not yet fully understood. It might be similar to PCV2, although the high level of genetic diversity between PCV2 and PCV3 may suggest unique pathogenesis and immune modulation paths for the novel PCVs [94]. Due to the variability of symptoms that might correspond with PCV2 infection, the clinical manifestation of PCV2 infection is currently referred to as PCVAD. Five clinical forms of PCVAD can be distinguished: PCV2 subclinical infection (PCV2-SI), PCV2 systemic disease (PCV2-SD), PCV2 lung disease (PCV2-LD), PCV2 enteric disease (PCV2-ED) and PCV2 reproductive disease (PCV2-RD) [95]. PCV2-SD is most common in pigs between 5 and 12 weeks of age and is associated with clinical symptoms such as weight loss, dyspnoea, cough, pale skin or jaundice, diarrhea and nervous system disorders [95]. PCV2-LD causes respiratory distress, inhibition of daily weight gain, loss of appetite and fever. For PCV2-ED, diarrhea is reported mainly in pigs between 8 and 16 weeks of age. In the reproductive form of PCV2 infection, the dominant symptoms are late abortions, giving birth to dead and mummified piglets, and repeated oestrus [95].

PCV3 has been associated with several clinical presentations [7,8,30,96]. It was detected in pigs demonstrating PDNS-like symptoms, reproductive failure [7] and respiratory symptoms [8] and in pigs with multisystemic inflammation, vasculitis and myocarditis [8.96]. Reproductive failure and multisystemic inflammation seem to be most consistently reported across the current literature [7,8,30,97].

Jiang et al. (2019) showed PCV3 fulfilled Koch’s postulates and could cause PDNS in piglets. Robert Koch established his famous postulates in the 19^th^ century as stringent guidelines to evaluate causation in infectious disease. These original postulates require isolation of the assumed pathogen and then reinfection of the healthy host to prove causation [98]. Jiang et al. (2019) inoculated 4- and 8-week-old SPF piglets and demonstrated the first successful reproduction of PDNS in animals that were infected with PCV3 or PCV3 with keyhole limpet hemocyanin (KLH) immunostimulant. All five 4-week-old PCV3-inoculated piglets presented following symptoms: fever, anorexia, coughing, sneezing, diarrhea, lethargy, rubefaction on the skin and the ears, multifocal papules, shivering and/or hyperspasmia. Two piglets in this group inoculated with PCV3 developed severe clinical signs, cardiac pathologies and multisystemic inflammation and died during the experiment. In addition, typical clinical signs of PDNS were observed in 8-week-old piglets inoculated with PCV3, although all the animals survived the entire period of the study (28 days). Histopathological lesions included extensive lymphocyte necrosis and depletion, necrotic epithelial cells and infiltration of inflammatory cells (neutrophils/histiocytes, macrophages, eosinophils), together with secondary suppurative bronchopneumonia with inflammatory exudates. Various tissues and organs (lung, heart, kidney, lymph nodes, spleen, liver and small intestines) of PCV3-inoculated piglets were abundant in PCV3-positive cells detected by immunohistochemical staining. The study confirms the role of PCV3 as the etiological agent of PDNS. The reproduction of PDNS by infection of PCV3 alone suggests PCV3 is more pathogenic for piglets than PCV2 [99]. The peak of viremia (approximately 7.72 × 10^8^ PCV3 copies/mL) in the 4-week-old PCV3-inoculated piglets was detected at 21 DPI. The highest level of PCV3 (7.92 × 10^7^ copies/mL) in the 8-week-old PCV3-inoculated piglets was also detected at 21 DPI. Seroconversion to PCV3 antibodies was observed in both the 4- and 8-week-old PCV3-inoculated piglets at 14 DPI. The highest amount of viral DNA was seen in sera correlated with a low titer of anti-PCV3 antibodies [99].

In 2020, Mora-Diaz reported in vivo replication of PCV3 in 6-week-old caesarean-derived, colostrum-deprived (CD/CD) pigs under experimental conditions. Eight pigs were inoculated with PCV3 isolate ISU27734 (6.6 × 10^10^ copies/mL) and stimulated by KLH. PCV3 was isolated from three diagnostic cases submitted to the Iowa State University Veterinary Diagnostic Laboratory from three different sites reporting weak-born pigs or elevated stillborn and mummified fetuses. The virus was isolated from multiple tissues, including the lung, kidney, heart and brain, of perinatal pigs with encephalitis and/or myocarditis and stillborn and mummified fetuses. Although all animals infected with the PCV3 isolate remained clinically healthy throughout the study, histological evaluation showed lesions of ongoing multisystemic inflammation, lymphoplasmacytic myocarditis and periarteritis (4/8 pigs). PCV3 replication was confirmed by in situ hybridization (ISH) within myocardiocytes, the tunica media and endothelial cells of arteries, and inflammatory cells. Lymphoplasmacytic interstitial nephritis and periarteritis in kidney tissue and lymphoplasmacytic periarteritis and arteritis of the intestinal serosa of PCV3- inoculated pigs were demonstrated. PCV3 replication was also confirmed within inflammatory cells, the tubular renal epithelium, endothelial cells and the tunica media of arteries. Viremia in CD/CD pigs, first detected at 7 dpi, was detected in all animals by 28 dpi. IgM antibody response was detected in the range of 7–14 dpi in 5/8 PCV3-inoculated pigs, but no IgG seroconversion was detected throughout the study [100].

In another study, PCV3 genetic material was detected by ISH in four pigs with thrown-back ears, wasting symptoms and rough hair [101]. During necropsy, tissue samples (superficial inguinal and submandibular lymph nodes, Peyer’s patches, tonsils, hearts, cerebra, cerebella and pons, kidneys, lungs, spleens, large and small bowels, livers and nasal turbinates) were collected from these animals to perform a histopathological examination and to determinate PCV3, PCV2 and/or PRRSV. PCV3 was detected in the following structures: lymph nodes (2/3 specimens), tonsils (2/3), Peyer’s patches (1/3), white pulp of the spleen (3/4), myocardiocyte-like cells (4/4), white matter (4/4), gray matter (4/4), renal tubular epithelial cells (2/4), lung (2/4), Kupffer’s cells (3/4), intestinal smooth muscle-like cells of the small bowel and spiral colon (1/4) and the nasal turbinate (2/2). Histopathological examination revealed multi-organic lymphoplasmacytic periarteritis and arteritis in the following structures: mesenteric arteries, heart kidneys, spleen, arterioles portal, meninges, lungs and/or stomach. Other changes that were observed were lymphoplasmacytic myocarditis (4/4), mild lymphoplasmacytic and histiocytic interstitial pneumonia (1/4), mild lymphoplasmacytic nephritis (3/4) and mild lymphoplasmacytic periportal hepatitis (3/4). Exclusion of PCV2 and PRRSV and overlapping of PCV3 sites with lesions may indicate the pathogenic effects of PCV3 [101].

In Temeeyasen et al. (2021), pigs remained clinically unremarkable during the entire experiment; however, in two (50%) out of four pigs inoculated with PCV3 and three (75%) out of four pigs inoculated with PCV3 and KLH, histologic lesions in multiple tissues (brain, heart, liver, spleen, kidney, small intestine) were found. These lesions were characteristic of multisystemic inflammation and included lymphoplasmacytic encephalitis with perivasculitis, random multifocal lymphoplasmacytic hepatitis, lymphoplasmacytic interstitial nephritis and periarteritis, and lymphoplasmacytic myocarditis and periarteritis [96]. Chronic viremia appeared in experimentally infected CD/CD pigs at 3 DPI and was detectable until the end of the experiment (42 DPI). Pigs were inoculated with PCV3 only or with PCV3 and KLH. In the first group, the IgG level reached a maximum at 17 DPI, then decreased rapidly by 24 DPI and remained at the positive level until the end of the experiment. Conversely, in the second group, the IgG response was prolonged. Antibodies were detected from 10 DPI, reached a maximum level at 28 DPI, and then the IgG level decreased slightly, remaining detectable until the end of the experiment (42 DPI). There are still numerous gaps in knowledge concerning the mode of action attributed to PCV3 neutralizing antibodies [96].

Recently, Vargas-Bermudez et al. (2021) conducted research in a Colombian pig farm, where an increase in pregnant sow mortality and the number of stillborn piglets, abortions, birth of weak piglets and decrease in farrowing rate were observed [102]. Serum samples from 10 gilts and 40 sows were collected and tested for PCV3, PCV2, porcine parvovirus (PPV) 1 and 7, atypical porcine pestivirus (APPV) and porcine reproductive and respiratory syndrome virus (PRRSV). Four gilts and six sows (10/50; 20%) were positive for PCV3. The gilt with a PCV3 viral titer in the pre-farrowing serum of 7.4 × 10^3^ (5.8 log copies (lgc)/mL) was selected for further observation. During the delivery of 10 piglets, dystocia and mummified fetuses appeared. Two of the piglets died a few hours after delivery, previously showing respiratory symptoms and an inability to suckle. The third piglet died at 3 weeks of age, and another was euthanized at 5 weeks of age; both presented waste symptoms. From piglets with waste symptoms, samples of spleen, mesenteric lymph nodes, tonsils, liver, kidney, heart and lung were collected. Other animals from this litter were sampled for sera from birth to week six, including precolostrum sera. Samples from gilts included pre-farrowing serum, post farrowing serum, nasal swab, vaginal swab, colostrum, placenta and mummified fetus. PCV3 was confirmed in all samples taken. The viral titers in the placenta achieved 2 × 10^5^ (7.23 lgc/mL), and 7.5 × 10^9^ (11.8 lgc/mL) in the mummified fetus. In the two piglets that died shortly after delivery, the viral load in the serum was 5.42 × 10^6^ and 1 × 10^7^ (8.64 and 8.93 lgc/mL), and high levels were noted in tissues such as lymph nodes and lungs: (5.19 × 10^4^) (6.64 lgc/mL) and (7.7 × 107) (9.81 lgc/mL), respectively. Placental necrosis and inflammation, lymphoid depletion and granuloma formation in the mesenteric lymph node, moderate multifocal thickening of alveolar septa in the lungs and kidney interstitial nephritis were found in the microscopic examination of postpartum and dead piglets’ tissues. The high tropism of the virus to the placental tissue explains the reproductive signs in infected sows and vertical transmission [102].

Immunosuppression and secondary infection occurrence are associated with PCV3 infections, which confirms the study by Mai et al. (2021) on PPV and PCV3 co-infections [103]. A total of 209 serum samples (105 PCV3-positive, 104 PCV3-negative) from commercial swine herds, 62 PCV3-postive sow serum samples, and 20 PCV3-positive aborted fetuses (from farms with or without reproductive failure) were selected for the study. The results revealed that the PPV positive rate was significantly higher in PCV3-positive serum (33/105 in PCV3 positive samples and 15/104 in PCV3 negative samples) and in PCV3-positive serum from pigs with reproductive failure (RF) (20/39 in PCV3-positive RF serum and 8/23 in PCV3-positive non-RF serum) as well as in PCV3-positive aborted fetus samples (11/20). Higher PCV3 viral loads in PPV7-positive samples compared with PPV7-negative samples were also observed [103].

The clinical symptoms, anatomopathological changes, viral load in serum and lesions, humoral immunity response and interaction with other pathogens in the course of PCV3 infection in pigs indicate that PCV3 is pathogenic for pigs and can cause economic losses in the swine industry. Saporiti et al. (2021) proposed to distinguish two major disease outcomes related with PCV3 infection: PCV3-reproductive disease (PCV3-RD) in sows and fetuses/neonatal piglets and PCV3-systemic disease (PCV3-SD) in pre- and post-weaning pigs. The diagnosis of PCV3-RD would be made after meeting the following criteria: late reproductive problems and higher perinatal mortality, multisystemic lymphoplasmacytic to lymphohistiocytic perivascular inflammation and moderate to high amount of PCV3 genomic DNA in damaged tissues, while PCV3-SD diagnosis would be based on the presence of weight loss, rough hair, neurological signs, multisystemic lymphoplasmacytic to lymphohistiocytic perivascular inflammation and a moderate to high amount of PCV3 genome in damaged tissues [104].

On the contrary, there are numerous reports on the asymptomatic course of PCV3 infection in pigs [20,105,106,107]. In the study of Zheng et al. (2017), 59.46% (*n* = 222) of samples (including hearts, livers, lungs, kidneys, spleens and umbilical cords) from 37 natural stillborn fetuses were PCV3 positive, although there were no clinical signs of infection in either multiparous sows or live-born infants [107]. In another study, PCV3 DNA was detected in 21.9% (*n* = 105) of clinically healthy pigs [106]. It is worth emphasizing that no significant clinical signs were observed in the studies conducted by Temeeyasen et al. and Mora-Diaz et al.; however, histological lesions consistent with multi-systemic inflammation were observed in investigated pigs [96,100],

To date, the pathogenicity, pathology and immunity related to PCV4 infection remain unknown. In the study by Zhang (2020), pigs positive for PCV4 DNA had severe clinical signs, including signs of PDNS, respiratory symptoms and enteritis, although some of them were clinically healthy. PCV4 caused monoinfection or co-infection with PRRSV or PCV2. The coinfection with these pathogens or other undetected viral or bacterial agents can influence the disease process and clinical outcomes [9]. The selected parameters characteristics for porcine circoviruses are presented in Table 1.

## 7. Development of Vaccine against New PCVs—Current Data

A commercial vaccine against PCV2 has been available since 2006 and is effective in preventing losses associated with PCVAD and clinical disease through decreasing the viremia, virus shedding and lesions in affected pigs [111]. The stimulation of humoral and cell-mediated immunity through strong neutralizing antibody responses against PCV2 Cap and IFN-γ secreting is crucial to elicit the appropriate vaccine-mediated host response [94]. PCV2a Cap is included in most commercially available vaccines and provides protection, regardless of the subtype, due to cross-reactivity. Subunit vaccines containing PCV2 Cap protein expressed by baculovirus and inactivated chimeric PCV1-2 vaccines against this pathogen are available [94]. Nevertheless, vaccination failure occasionally occurs, and vaccination programs are not completely capable of eliminating PCV2 circulation in herds [117]. Known technology options for PCV2 vaccine design could form the basis of PCV3 vaccine development. The low sequence similarity of the PCV3 genome to other PCV genotypes and the lack of cross-reaction associated with PCV1 and PCV2 according to some authors make PCV2 vaccines ineffective against PCV3 infections [118]. PCV3 Cap and replicase proteins association with PCV2 is scarce (37%) [7]. Interestingly, according to Woźniak et al. [111], the omission of anti-PCV2 vaccination favored a greater prevalence of PCV3 in the pig serum compared to animals from farms with vaccination programs.

To date, research related to vaccine design is at the early stage, although studies on the design of monoclonal antibodies (mABs) against some PCV3 proteins, epitopes recognition and the expression of antigens in vitro have been conducted, numerous aspects regarding immune response and virus characteristics remain unknown. The results obtained create the basis for vaccine design and the development of other products based on similar technologies.

The replicase and Cap, the major proteins of PCV3, are both immunogenic [94], although Cap is sufficient and necessary for protection in the case of PCV2. Capsid protein, which induces neutralizing antibodies, is a main target for serological diagnostic tests together with vaccine design [119]. The detailed structure of PCV3 Cap has not yet been described. Efficient expression of Cap with a minimal financial effort is desirable. In vitro expressed strains would be beneficial in better understanding the PCV3 and/or PCV4 infection pathomechanism, structure and expression, especially in Prokaryotes. The expression system of *E. coli* is believed to be the most efficient [119]. The anterior domain of Cap contains rare codons that limit gene expression in *E. coli* [119,120]. However, deletion of repetitive arginine residues located in the anterior region of the N-terminal nuclear location signal (NLS) domain of the PCV3 Cap did not affect the virus-like particles (VLPs) assembling and, additionally, increased the level of PCV3 protein expression in *E. coli* [120]. In view of the lack of an efficient cultivation method of PCV3 and PCV4, some expectations for vaccine development are placed on VLPs, which are morphologically and immunologically similar to the native viruses. In vitro assembling of PCV3 VLPs may lead to the design of an effective vaccine, owing to their ability to induce strong immune response [121]. PCV3 Cap is capable of self-assembly into VLPs [120]. The vaccine based on self-assembled VLPs from PCV2 Cap was characterized by better prophylactic efficacy compared to vaccines containing inactivated PCV2 viruses [122,123,124]. A similar vaccine may prove effective against PCV3. Bi et al. (2020) reported the strategies to obtain high-quality PCV3 VLPs using the prokaryotic *E. coli* expression system. Produced VLPs contained type-specific surface epitopes, different for various PCVs genotypes [125]. In 2021, Wang et al. successfully expressed the PCV4 Cap in an *E. coli* expression system, which was capable of self-assembling into VLPs. Produced VLPs had high antigenicity, although the cross-reaction conferred by humoral immune responses between PCV4 and PCV3 or PCV2 was unlikely in this study. The predicted PCV4 immune epitopes had significantly low identity to PCV3 or PCV2 [119].

Importantly, a previous study reports successful development of mABs that recognize the PCV3 Cap protein [126]. Epitope information plays a pivotal role in understanding and controlling the virus and thus differentiation of mABs, useful in rational vaccine design. Jiang et al. [14] identified three linear B cell epitopes recognized by mABs against the PCV3 Cap protein: ^57^NKPWH^61^, ^140^KHSRYFT^146^ and ^161^QSLFFF^166^. They also showed that ^140^KHSRYFT^146^ has some characteristics of a decoy epitope and that such motifs are present in all known species of PCVs. The authors suggested that it may be a common sign and significant tool to evade the host’s immune response [14]. Anti-PCV3 monoclonal antibodies and PCV3 VLPs are valuable for basic PCV3 research as well as VLP-based prophylactic vaccine and diagnostic tool development.

Peswani et al. [127] tested a chimeric protein consisting of antigens from PCV2d and PCV3 capsid proteins. The PCV2d-based recombinant protein from their protocol did not form VLPs. Despite this, it could effectively induce capsid-specific and PCV2d-neutralizing antibodies. They generated recombinant PCV2d capsid proteins from *E. coli* expression, and the resulting protein could induce neutralizing antibodies in test animals, suggesting its potential as a vaccine against PCV2d. Interestingly, the chimera is almost quantitatively cleaved in the linker sequence to release high quantities of PCV2d and measurable levels of PCV3. Detectable levels of peptides derived from PCV3 capsid proteins suggest that this process can be adapted to produce PCV3 vaccines. However, the only identified peptide signals specific for PCV3 were present at low to very low signal-to-noise ratios. The authors suggest further optimization, involving prevention of proteolytic degradation and more effective fractionations, could possibly enrich PCV3-derived peptides along with the purified soluble PCV2d antigen [127].

Proper challenge models are also important to develop successful animal studies for vaccine licensure. In 2020, with the use of PCV3 circular DNA obtained in vitro, SPF Kunming mice were infected with PCV3 infectious clones without the necessity of exogenous expression vectors. Five out of ten experimental mice received rescued PCV3 virus. The investigated strain was capable of infecting the myocardium and lung tissue of mice. Anti-PCV3 Cap mABs were also obtained. The study used a 3D4/21 cell line for replicating and packaging the virus, limiting the possibility of contamination by the PCV1 genome that may be present in other cell lines, e.g., the PK-15 cell line, previously used for packaging and rescue of the virus. The results of this study could be useful in the future for the development of subunit vaccines against PCV3 or mABs drugs [128].

## 8. Conclusions

A wide variety of clinical symptoms have been reported in pigs from which PCV3 and PCV4 were isolated. PCV3, although described relatively recently, is very widely distributed in the world and is characterized by high prevalence. Given the ease of spreading, potential high evolutionary rate and possible harm to the pig industry, the introduction of effective specific immunoprophylaxis and prevention of widespread novel PCVs is desirable. A vaccine against PCV3 is not available yet, and research is at the early stages; however, several important issues have been discovered. Pathogenicity, structure and immunity related to PCV4 are still not fully understood.

## Figures and Tables

**Table 1:** Selected parameters characteristics for porcine circoviruses.

Species	PCV1	PCV2	PCV3	PCV4	References
**Year of** **description**	1974	1998	2016	2019	[5,6,7,8,9]
**Mortality in pigs**	Not reported	Weaners: 11.2% (1.4–50%) Finisher pigs: 5.2%	Up to 7th day of life: 40%, Later: 16% within 6 months	Unknown	[12,21,108]
**Pathogenicity** **for pigs**	no	Yes	yes	Unknown	[6,7,8,9,10,12,85]
**Seroconversion**	7 dpi	7 dpi	IgG 3 dpi IgM 7 dpi	Unknown	[12,96,100,109]
**Humoral immunity duration**	Up to 39–47 weeks pi	23 weeks	IgM up to 28 dpi IgG up to 42 dpi	Unknown	[12,96,100,110]
**Vaccine**	No vaccine needed	Subunit; chimeric PCV1-2 vaccines	Not available (work in progress)	Not available	[12,111]
**Occurrence**	Worldwide	Worldwide	Worldwide	China, South Korea	[7,9,16,18,30,46,52,111,112,113]
**World prevalence**	Low	High	High	Unknown	[30,114,115]
**Other species with detectable virus**	Wild boars	Humans Wild boars Mice Cattle Racoon Dogs Foxes Minks Flies Mosquitoes	Wild boars Ticks Donkeys Cattle Dogs Mice Baboons Wild ruminants (e.g., red deer, fallow deer, mouflon)	Unknown	[40,64,65,66,67,68,69,70,71,72,79,81,82,85,89,116]
